# (*E*)-1-(2-Methyl-4-phenyl­quinolin-3-yl)-3-phenyl­prop-2-en-1-one

**DOI:** 10.1107/S1600536811007057

**Published:** 2011-03-02

**Authors:** Wan-Sin Loh, Hoong-Kun Fun, R. Prasath, S. Sarveswari, V. Vijayakumar

**Affiliations:** a X-ray Crystallography Unit, School of Physics, Universiti Sains Malaysia, 11800 USM, Penang, Malaysia; b Organic Chemistry Division, School of Advanced Sciences, VIT University, Vellore 632 014, India

## Abstract

In the title compound, C_25_H_19_NO, the quinoline ring system is approximately planar, with a maximum deviation of 0.32 (1) Å, and forms dihedral angles of 80.74 (3) and 81.71 (4)° with the two phenyl rings. In the crystal. mol­ecules are stacked along the *b* axis by way of a C—H⋯π inter­action and a weak π–π inter­action between the pyridine and phenyl rings with a centroid–centroid distance of 3.6924 (5) Å.

## Related literature

For background to and the biological activity of quinoline derivatives, see: Morimoto *et al.* (1991 ▶); Michael (1997 ▶); Markees *et al.* (1970 ▶); Campbell *et al.* (1988) ▶; Maguire *et al.* (1994 ▶); Chen *et al.* (2001 ▶). For the biological activity of chalcones, see: Dimmock *et al.* (1999 ▶). For bond-length data, see: Allen *et al.* (1987 ▶). For related structures, see: Loh, Fun, Sarveswari *et al.* (2010 ▶); Loh, Fun, Viji *et al.* (2010 ▶); Shahani *et al.* (2010 ▶). For the stability of the temperature controller used in the data collection, see: Cosier & Glazer (1986 ▶).
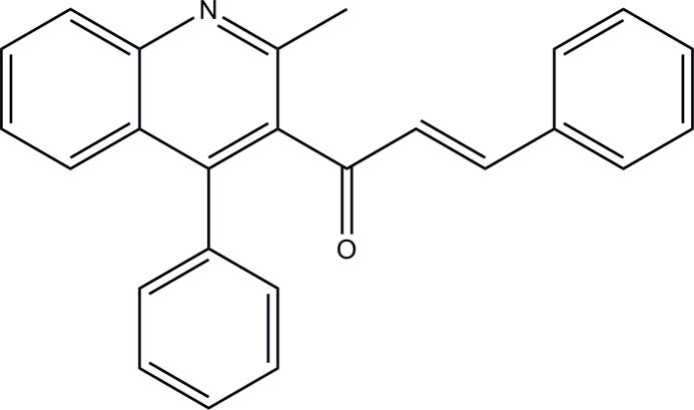


## Experimental

### 

#### Crystal data

C_25_H_19_NO
*M* *_r_* = 349.41
Monoclinic, 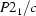

*a* = 10.5830 (5) Å
*b* = 10.2189 (5) Å
*c* = 18.4509 (7) Å
β = 114.147 (2)°
*V* = 1820.80 (14) Å^3^
*Z* = 4
Mo *K*α radiation
μ = 0.08 mm^−1^
*T* = 100 K
0.57 × 0.48 × 0.35 mm

#### Data collection

Bruker SMART APEXII DUO CCD area-detector diffractometer
Absorption correction: multi-scan (*SADABS*; Bruker, 2009 ▶) *T*_min_ = 0.957, *T*_max_ = 0.973
27166 measured reflections
6509 independent reflections
5734 reflections with *I* > 2σ(*I*)
*R*_int_ = 0.024

#### Refinement

*R*[*F* ^2^ > 2σ(*F* ^2^)] = 0.042
*wR*(*F* ^2^) = 0.125
*S* = 1.05
6509 reflections
245 parameters
H-atom parameters constrained
Δρ_max_ = 0.45 e Å^−3^
Δρ_min_ = −0.17 e Å^−3^

### 

Data collection: *APEX2* (Bruker, 2009 ▶); cell refinement: *SAINT* (Bruker, 2009 ▶); data reduction: *SAINT*; program(s) used to solve structure: *SHELXTL* (Sheldrick, 2008 ▶); program(s) used to refine structure: *SHELXTL*; molecular graphics: *SHELXTL*; software used to prepare material for publication: *SHELXTL* and *PLATON* (Spek, 2009 ▶).

## Supplementary Material

Crystal structure: contains datablocks global, I. DOI: 10.1107/S1600536811007057/is2680sup1.cif

Structure factors: contains datablocks I. DOI: 10.1107/S1600536811007057/is2680Isup2.hkl

Additional supplementary materials:  crystallographic information; 3D view; checkCIF report

## Figures and Tables

**Table 1:** Hydrogen-bond geometry (Å, °) *Cg*3 is the centroid of the C20–C25 phenyl ring.

*D*—H⋯*A*	*D*—H	H⋯*A*	*D*⋯*A*	*D*—H⋯*A*
C3—H3*A*⋯*Cg*3^i^	0.93	2.72	3.5265 (10)	146

Symmetry code: (i) 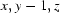.

**Fig. 1.**
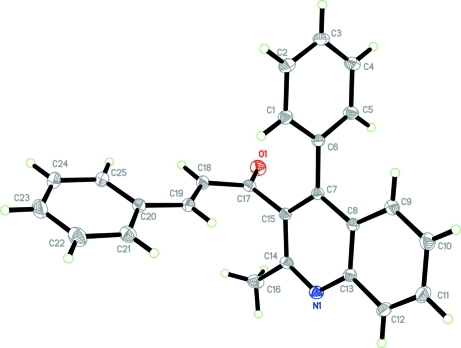
The molecular structure of the title compound, showing 50% probability displacement ellipsoids and the atom-numbering scheme.

**Fig. 2.**
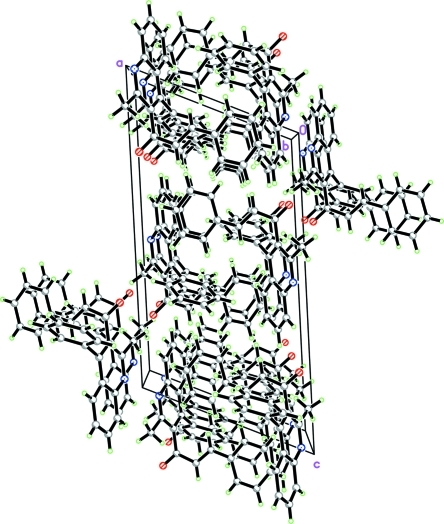
The crystal packing of the title compound, viewed along the b axis.

## supplementary crystallographic information

### Comment

The quinolines and their derivatives are very important compounds because of their wide occurrence in natural products (Morimoto *et al.*, 1991; Michael, 1997) and biologically active compounds (Markees *et al.*, 1970; Campbell *et al.*, 1988). A large variety of quinolines have interesting physiological activities and found to have attractive applications as pharmaceuticals, agrochemicals and as synthetic building blocks (Maguire *et al.*, 1994; Chen *et al.*, 2001). The chalcones are open chain flavonoids, possessed a variety of biological activities, including antioxidant, anti-inflammation, antimicrobial, antiprotozoal, antiulcer, as well as other properties (Dimmock *et al.*, 1999). In continuation of our interest in the synthesis of chalcone derivatives (Loh, Fun, Sarveswari *et al.* (2010); Loh, Fun, Viji *et al.* (2010); Shahani *et al.*, 2010), herein we report another new chalcone.

In the title compound (Fig. 1), the quinoline ring system (C7–C13/N1/C14/C15) is aproximately planar with a maximum deviation of 0.32 (1) Å at atom C15 and forms dihedral angles of 80.74 (3) and 81.71 (4)° with the C1–C6 and C20–C25 phenyl rings, respectively. Bond lengths (Allen *et al.*, 1987) and angles are within the normal ranges.

There are no significant intermolecular hydrogen bonds observed in the crystal packing (Fig. 2). The molecules are stacked along the *b* axis by way of a C–H···π interaction (Table 1) which involves the phenyl ring (C20–C25) and a weak aromatic π–π interaction between the pyridine (N1/C13/C8/C7/C15/C14; centroid *Cg*1) and phenyl (C8–C13; centroid *Cg*2) rings with a separation of *Cg*1···*Cg*2 being 3.6924 (5) Å.

### Experimental

A mixture of 3-acetyl-2-methyl-4-phenylquinoline (2.6 g, 0.01 *M*) and benzaldehyde (1.06 g, 0.01 *M*) and a catalytic amount of KOH in 40 ml of distilled ethanol was stirred for about 12 h. The resulting mixture was concentrated to remove ethanol and then poured onto ice and neutralized with distilled acetic acid. The resultant solid was filtered, dried and purified by column chromatography using 1:1 mixture of ethylacetate and petroleum ether. Recrystallization was done in (8:4) petroleum ether, acetone mixture (*m.p.*: 422–423 K, yield: 82%).

### Refinement

All H atoms were positioned geometrically (C—H = 0.93 or 0.96 Å) and were refined using a riding model, with *U*_iso_(H) = 1.2 or 1.5*U*_eq_(C). A rotating group model was applied to the methyl groups.

### Crystal data

**Table:**

C_25_H_19_NO	*F*(000) = 736
*M**_r_* = 349.41	*D*_x_ = 1.275 Mg m^−^^3^
Monoclinic, *P*2_1_/*c*	Mo *K*α radiation, λ = 0.71073 Å
Hall symbol: -P 2ybc	Cell parameters from 9875 reflections
*a* = 10.5830 (5) Å	θ = 2.9–35.1°
*b* = 10.2189 (5) Å	µ = 0.08 mm^−^^1^
*c* = 18.4509 (7) Å	*T* = 100 K
β = 114.147 (2)°	Block, colourless
*V* = 1820.80 (14) Å^3^	0.57 × 0.48 × 0.35 mm
*Z* = 4	

### Data collection

**Table:**

Bruker SMART APEXII DUO CCD area-detector diffractometer	6509 independent reflections
Radiation source: fine-focus sealed tube	5734 reflections with *I* > 2σ(*I*)
graphite	*R*_int_ = 0.024
φ and ω scans	θ_max_ = 32.5°, θ_min_ = 2.8°
Absorption correction: multi-scan (*SADABS*; Bruker, 2009)	*h* = −15→15
*T*_min_ = 0.957, *T*_max_ = 0.973	*k* = −15→15
27166 measured reflections	*l* = −27→27

### Refinement

**Table:**

Refinement on *F*^2^	Primary atom site location: structure-invariant direct methods
Least-squares matrix: full	Secondary atom site location: difference Fourier map
*R*[*F*^2^ > 2σ(*F*^2^)] = 0.042	Hydrogen site location: inferred from neighbouring sites
*wR*(*F*^2^) = 0.125	H-atom parameters constrained
*S* = 1.05	*w* = 1/[σ^2^(*F*_o_^2^) + (0.0699*P*)^2^ + 0.5003*P*] where *P* = (*F*_o_^2^ + 2*F*_c_^2^)/3
6509 reflections	(Δ/σ)_max_ = 0.001
245 parameters	Δρ_max_ = 0.45 e Å^−^^3^
0 restraints	Δρ_min_ = −0.17 e Å^−^^3^

### Special details

**Table:**

Experimental. The crystal was placed in the cold stream of an Oxford Cryosystems Cobra open-flow nitrogen cryostat (Cosier & Glazer, 1986) operating at 100.0 (1) K.
Geometry. All e.s.d.'s (except the e.s.d. in the dihedral angle between two l.s. planes) are estimated using the full covariance matrix. The cell e.s.d.'s are taken into account individually in the estimation of e.s.d.'s in distances, angles and torsion angles; correlations between e.s.d.'s in cell parameters are only used when they are defined by crystal symmetry. An approximate (isotropic) treatment of cell e.s.d.'s is used for estimating e.s.d.'s involving l.s. planes.
Refinement. Refinement of *F*^2^ against ALL reflections. The weighted *R*-factor *wR* and goodness of fit *S* are based on *F*^2^, conventional *R*-factors *R* are based on *F*, with *F* set to zero for negative *F*^2^. The threshold expression of *F*^2^ > σ(*F*^2^) is used only for calculating *R*-factors(gt) *etc*. and is not relevant to the choice of reflections for refinement. *R*-factors based on *F*^2^ are statistically about twice as large as those based on *F*, and *R*- factors based on ALL data will be even larger.

### Fractional atomic coordinates and isotropic or equivalent isotropic displacement parameters (Å^2^)

**Table:**

	*x*	*y*	*z*	*U*_iso_*/*U*_eq_	
O1	0.08254 (7)	0.61008 (7)	0.74003 (4)	0.02139 (13)	
N1	0.08585 (7)	0.73133 (7)	0.97579 (4)	0.01550 (13)	
C1	0.41109 (8)	0.42498 (8)	0.90617 (5)	0.01661 (14)	
H1A	0.4621	0.5004	0.9272	0.020*	
C2	0.47170 (8)	0.32137 (9)	0.88288 (5)	0.01897 (15)	
H2A	0.5635	0.3273	0.8895	0.023*	
C3	0.39571 (9)	0.20934 (8)	0.84990 (5)	0.01841 (15)	
H3A	0.4361	0.1406	0.8340	0.022*	
C4	0.25883 (9)	0.20050 (8)	0.84073 (5)	0.01956 (15)	
H4A	0.2073	0.1260	0.8182	0.023*	
C5	0.19882 (8)	0.30296 (8)	0.86519 (5)	0.01787 (15)	
H5A	0.1077	0.2959	0.8596	0.021*	
C6	0.27412 (8)	0.41613 (7)	0.89806 (4)	0.01322 (13)	
C7	0.20915 (7)	0.52474 (7)	0.92505 (4)	0.01297 (13)	
C8	0.19789 (7)	0.51611 (7)	0.99939 (5)	0.01355 (13)	
C9	0.24715 (8)	0.40797 (8)	1.05160 (5)	0.01717 (14)	
H9A	0.2860	0.3367	1.0369	0.021*	
C10	0.23797 (9)	0.40762 (9)	1.12373 (5)	0.02067 (16)	
H10A	0.2705	0.3361	1.1575	0.025*	
C11	0.17939 (9)	0.51514 (9)	1.14689 (5)	0.02056 (16)	
H11A	0.1737	0.5143	1.1959	0.025*	
C12	0.13073 (8)	0.62094 (8)	1.09741 (5)	0.01782 (15)	
H12A	0.0927	0.6916	1.1132	0.021*	
C13	0.13791 (7)	0.62353 (7)	1.02254 (5)	0.01411 (13)	
C14	0.09416 (8)	0.73623 (7)	0.90660 (5)	0.01470 (14)	
C15	0.15863 (7)	0.63547 (7)	0.87976 (4)	0.01344 (13)	
C16	0.02989 (9)	0.85248 (8)	0.85478 (5)	0.02108 (16)	
H16A	0.0019	0.9152	0.8841	0.032*	
H16B	−0.0495	0.8248	0.8089	0.032*	
H16C	0.0962	0.8918	0.8383	0.032*	
C17	0.17065 (8)	0.65370 (7)	0.80187 (5)	0.01473 (14)	
C18	0.28930 (8)	0.72866 (8)	0.80195 (5)	0.01588 (14)	
H18A	0.2986	0.7388	0.7543	0.019*	
C19	0.38512 (8)	0.78330 (7)	0.86795 (5)	0.01505 (14)	
H19A	0.3747	0.7692	0.9150	0.018*	
C20	0.50375 (8)	0.86236 (7)	0.87345 (5)	0.01548 (14)	
C21	0.59080 (9)	0.91308 (8)	0.94763 (5)	0.01973 (15)	
H21A	0.5724	0.8954	0.9918	0.024*	
C22	0.70455 (9)	0.98962 (9)	0.95593 (6)	0.02567 (19)	
H22A	0.7613	1.0235	1.0054	0.031*	
C23	0.73351 (10)	1.01549 (9)	0.89041 (7)	0.02676 (19)	
H23A	0.8094	1.0669	0.8959	0.032*	
C24	0.64859 (10)	0.96426 (9)	0.81659 (6)	0.02460 (18)	
H24A	0.6686	0.9807	0.7728	0.030*	
C25	0.53413 (9)	0.88875 (8)	0.80779 (5)	0.01951 (15)	
H25A	0.4774	0.8556	0.7581	0.023*	

### Atomic displacement parameters (Å^2^)

**Table:**

	*U*^11^	*U*^22^	*U*^33^	*U*^12^	*U*^13^	*U*^23^
O1	0.0222 (3)	0.0224 (3)	0.0164 (3)	−0.0036 (2)	0.0046 (2)	−0.0036 (2)
N1	0.0142 (3)	0.0149 (3)	0.0177 (3)	0.0010 (2)	0.0069 (2)	−0.0011 (2)
C1	0.0147 (3)	0.0170 (3)	0.0190 (3)	−0.0005 (2)	0.0079 (3)	−0.0020 (3)
C2	0.0164 (3)	0.0214 (4)	0.0209 (4)	0.0032 (3)	0.0095 (3)	−0.0004 (3)
C3	0.0228 (4)	0.0170 (3)	0.0172 (3)	0.0055 (3)	0.0099 (3)	0.0002 (3)
C4	0.0227 (4)	0.0153 (3)	0.0216 (4)	−0.0007 (3)	0.0100 (3)	−0.0044 (3)
C5	0.0163 (3)	0.0165 (3)	0.0216 (4)	−0.0016 (3)	0.0085 (3)	−0.0045 (3)
C6	0.0141 (3)	0.0129 (3)	0.0133 (3)	0.0012 (2)	0.0063 (2)	0.0000 (2)
C7	0.0123 (3)	0.0124 (3)	0.0147 (3)	−0.0005 (2)	0.0059 (2)	−0.0013 (2)
C8	0.0128 (3)	0.0130 (3)	0.0154 (3)	−0.0009 (2)	0.0064 (2)	−0.0007 (2)
C9	0.0201 (3)	0.0146 (3)	0.0174 (3)	0.0008 (3)	0.0083 (3)	0.0013 (3)
C10	0.0257 (4)	0.0188 (4)	0.0178 (4)	−0.0002 (3)	0.0093 (3)	0.0028 (3)
C11	0.0243 (4)	0.0231 (4)	0.0171 (3)	−0.0025 (3)	0.0114 (3)	−0.0006 (3)
C12	0.0184 (3)	0.0198 (3)	0.0182 (3)	−0.0017 (3)	0.0106 (3)	−0.0027 (3)
C13	0.0125 (3)	0.0145 (3)	0.0162 (3)	−0.0010 (2)	0.0068 (2)	−0.0016 (2)
C14	0.0139 (3)	0.0130 (3)	0.0165 (3)	0.0007 (2)	0.0054 (2)	−0.0008 (2)
C15	0.0131 (3)	0.0127 (3)	0.0146 (3)	−0.0005 (2)	0.0057 (2)	−0.0008 (2)
C16	0.0247 (4)	0.0167 (3)	0.0204 (4)	0.0068 (3)	0.0077 (3)	0.0025 (3)
C17	0.0165 (3)	0.0125 (3)	0.0148 (3)	0.0011 (2)	0.0061 (3)	0.0000 (2)
C18	0.0188 (3)	0.0154 (3)	0.0144 (3)	−0.0010 (2)	0.0078 (3)	0.0007 (2)
C19	0.0166 (3)	0.0146 (3)	0.0150 (3)	0.0001 (2)	0.0075 (3)	0.0007 (2)
C20	0.0163 (3)	0.0130 (3)	0.0180 (3)	0.0005 (2)	0.0078 (3)	0.0006 (2)
C21	0.0189 (3)	0.0182 (3)	0.0204 (4)	−0.0008 (3)	0.0064 (3)	−0.0012 (3)
C22	0.0199 (4)	0.0217 (4)	0.0310 (5)	−0.0037 (3)	0.0060 (3)	−0.0046 (3)
C23	0.0211 (4)	0.0192 (4)	0.0429 (6)	−0.0039 (3)	0.0161 (4)	−0.0020 (4)
C24	0.0265 (4)	0.0203 (4)	0.0351 (5)	−0.0021 (3)	0.0209 (4)	0.0006 (3)
C25	0.0228 (4)	0.0178 (3)	0.0220 (4)	−0.0016 (3)	0.0133 (3)	−0.0005 (3)

### Geometric parameters (Å, °)

**Table:**

O1—C17	1.2244 (10)	C12—C13	1.4143 (11)
N1—C14	1.3159 (10)	C12—H12A	0.9300
N1—C13	1.3683 (10)	C14—C15	1.4309 (10)
C1—C2	1.3939 (11)	C14—C16	1.5020 (11)
C1—C6	1.3980 (10)	C15—C17	1.5054 (11)
C1—H1A	0.9300	C16—H16A	0.9600
C2—C3	1.3882 (12)	C16—H16B	0.9600
C2—H2A	0.9300	C16—H16C	0.9600
C3—C4	1.3910 (12)	C17—C18	1.4704 (11)
C3—H3A	0.9300	C18—C19	1.3464 (11)
C4—C5	1.3920 (11)	C18—H18A	0.9300
C4—H4A	0.9300	C19—C20	1.4611 (11)
C5—C6	1.3946 (11)	C19—H19A	0.9300
C5—H5A	0.9300	C20—C21	1.3997 (12)
C6—C7	1.4942 (10)	C20—C25	1.4009 (11)
C7—C15	1.3781 (10)	C21—C22	1.3905 (12)
C7—C8	1.4272 (11)	C21—H21A	0.9300
C8—C9	1.4178 (11)	C22—C23	1.3887 (15)
C8—C13	1.4186 (10)	C22—H22A	0.9300
C9—C10	1.3734 (12)	C23—C24	1.3912 (15)
C9—H9A	0.9300	C23—H23A	0.9300
C10—C11	1.4110 (13)	C24—C25	1.3886 (12)
C10—H10A	0.9300	C24—H24A	0.9300
C11—C12	1.3723 (12)	C25—H25A	0.9300
C11—H11A	0.9300		
			
C14—N1—C13	118.22 (7)	N1—C14—C15	122.61 (7)
C2—C1—C6	120.38 (7)	N1—C14—C16	117.07 (7)
C2—C1—H1A	119.8	C15—C14—C16	120.31 (7)
C6—C1—H1A	119.8	C7—C15—C14	120.18 (7)
C3—C2—C1	120.29 (7)	C7—C15—C17	121.14 (7)
C3—C2—H2A	119.9	C14—C15—C17	118.68 (6)
C1—C2—H2A	119.9	C14—C16—H16A	109.5
C2—C3—C4	119.64 (7)	C14—C16—H16B	109.5
C2—C3—H3A	120.2	H16A—C16—H16B	109.5
C4—C3—H3A	120.2	C14—C16—H16C	109.5
C3—C4—C5	120.16 (8)	H16A—C16—H16C	109.5
C3—C4—H4A	119.9	H16B—C16—H16C	109.5
C5—C4—H4A	119.9	O1—C17—C18	121.00 (7)
C4—C5—C6	120.62 (7)	O1—C17—C15	121.00 (7)
C4—C5—H5A	119.7	C18—C17—C15	117.98 (6)
C6—C5—H5A	119.7	C19—C18—C17	122.95 (7)
C5—C6—C1	118.89 (7)	C19—C18—H18A	118.5
C5—C6—C7	120.13 (7)	C17—C18—H18A	118.5
C1—C6—C7	120.97 (7)	C18—C19—C20	126.87 (7)
C15—C7—C8	117.99 (7)	C18—C19—H19A	116.6
C15—C7—C6	121.67 (7)	C20—C19—H19A	116.6
C8—C7—C6	120.34 (6)	C21—C20—C25	118.83 (7)
C9—C8—C13	118.94 (7)	C21—C20—C19	118.42 (7)
C9—C8—C7	123.30 (7)	C25—C20—C19	122.75 (7)
C13—C8—C7	117.72 (7)	C22—C21—C20	120.66 (8)
C10—C9—C8	120.55 (7)	C22—C21—H21A	119.7
C10—C9—H9A	119.7	C20—C21—H21A	119.7
C8—C9—H9A	119.7	C23—C22—C21	120.04 (9)
C9—C10—C11	120.36 (8)	C23—C22—H22A	120.0
C9—C10—H10A	119.8	C21—C22—H22A	120.0
C11—C10—H10A	119.8	C22—C23—C24	119.77 (8)
C12—C11—C10	120.25 (8)	C22—C23—H23A	120.1
C12—C11—H11A	119.9	C24—C23—H23A	120.1
C10—C11—H11A	119.9	C25—C24—C23	120.45 (9)
C11—C12—C13	120.61 (8)	C25—C24—H24A	119.8
C11—C12—H12A	119.7	C23—C24—H24A	119.8
C13—C12—H12A	119.7	C24—C25—C20	120.25 (8)
N1—C13—C12	117.53 (7)	C24—C25—H25A	119.9
N1—C13—C8	123.19 (7)	C20—C25—H25A	119.9
C12—C13—C8	119.28 (7)		
			
C6—C1—C2—C3	1.30 (13)	C7—C8—C13—C12	−177.19 (7)
C1—C2—C3—C4	−0.49 (13)	C13—N1—C14—C15	−1.81 (11)
C2—C3—C4—C5	−0.60 (13)	C13—N1—C14—C16	177.03 (7)
C3—C4—C5—C6	0.90 (13)	C8—C7—C15—C14	−1.49 (11)
C4—C5—C6—C1	−0.10 (12)	C6—C7—C15—C14	178.55 (7)
C4—C5—C6—C7	−179.22 (8)	C8—C7—C15—C17	178.10 (6)
C2—C1—C6—C5	−1.00 (12)	C6—C7—C15—C17	−1.87 (11)
C2—C1—C6—C7	178.11 (7)	N1—C14—C15—C7	3.21 (11)
C5—C6—C7—C15	−101.19 (9)	C16—C14—C15—C7	−175.58 (7)
C1—C6—C7—C15	79.71 (10)	N1—C14—C15—C17	−176.38 (7)
C5—C6—C7—C8	78.85 (10)	C16—C14—C15—C17	4.83 (11)
C1—C6—C7—C8	−100.25 (9)	C7—C15—C17—O1	86.75 (10)
C15—C7—C8—C9	−179.35 (7)	C14—C15—C17—O1	−93.66 (9)
C6—C7—C8—C9	0.62 (11)	C7—C15—C17—C18	−94.95 (9)
C15—C7—C8—C13	−1.28 (10)	C14—C15—C17—C18	84.63 (9)
C6—C7—C8—C13	178.68 (6)	O1—C17—C18—C19	176.95 (8)
C13—C8—C9—C10	−0.51 (12)	C15—C17—C18—C19	−1.35 (11)
C7—C8—C9—C10	177.53 (7)	C17—C18—C19—C20	−178.06 (7)
C8—C9—C10—C11	−0.06 (13)	C18—C19—C20—C21	178.74 (8)
C9—C10—C11—C12	0.17 (13)	C18—C19—C20—C25	−1.61 (13)
C10—C11—C12—C13	0.30 (13)	C25—C20—C21—C22	0.75 (12)
C14—N1—C13—C12	178.75 (7)	C19—C20—C21—C22	−179.58 (8)
C14—N1—C13—C8	−1.20 (11)	C20—C21—C22—C23	−0.58 (14)
C11—C12—C13—N1	179.18 (8)	C21—C22—C23—C24	−0.19 (14)
C11—C12—C13—C8	−0.87 (12)	C22—C23—C24—C25	0.78 (14)
C9—C8—C13—N1	−179.09 (7)	C23—C24—C25—C20	−0.60 (14)
C7—C8—C13—N1	2.76 (11)	C21—C20—C25—C24	−0.16 (12)
C9—C8—C13—C12	0.96 (11)	C19—C20—C25—C24	−179.82 (8)

### Hydrogen-bond geometry (Å, °)

**Table:**

*Cg*3 is the centroid of the C20–C25 phenyl ring.

**Table:**

*D*—H···*A*	*D*—H	H···*A*	*D*···*A*	*D*—H···*A*
C3—H3A···Cg3^i^	0.93	2.72	3.5265 (10)	146

Symmetry codes: (i) *x*, *y*−1, *z*.
